# Alpha Particle Emitter Radiolabeled Antibodies in Cancer Therapy: Current Status, Challenges, and Future Prospects

**DOI:** 10.3390/ph18091316

**Published:** 2025-09-02

**Authors:** Citra R. A. P. Palangka, Isa Mahendra, Rien Ritawidya, Naoya Kondo, Takahito Nakajima

**Affiliations:** 1 Fundamental Technology Development Division, Near InfraRed Photo-Immunotherapy Institute, Kansai Medical University, Hirakata 573-1010, Japan; kondon@hirakata.kmu.ac.jp; 2 Department of Diagnostic and Interventional Radiology, Graduate School of Comprehensive Human Sciences, University of Tsukuba, Tsukuba 305-8577, Japan; isa.mahendra@brin.go.id (I.M.);; 3 Research Center for Radioisotope, Radiopharmaceutical, and Biodosimetry Technology, Research Organization for Nuclear Energy-National Research and Innovation Agency, BRIN, Puspiptek Area, South Tangerang 15314, Indonesia; rien001@brin.go.id

**Keywords:** alpha particle, radioimmunotherapy, monoclonal antibody

## Abstract

The utilization of antibodies to target radionuclides, known as radioimmunotherapy (RIT), has been actively researched for nearly five decades. Numerous significant preclinical and clinical studies in cancer therapy have been highlighted. Among them, RIT using alpha-emitting nuclides has shown high effectiveness and has attracted much interest in recent years. This review presents an overview of the basic elements of alpha-RIT, namely the target proteins (monoclonal antibodies and antibody-derived proteins), alpha-emitting radionuclides, and labeling methods, which are currently being adapted in cancer therapy. It also highlights efforts to expand the potential of alpha-RIT, including the control of radioactivity distribution in the body.

## 1. Introduction

The exploration of radionuclides conjugated with antibodies commenced in the early 1950s, building upon a concept envisioned by Erlich in 1900 known as the “magic bullet” [1]. This innovative approach, called radioimmunotherapy (RIT), combined the therapeutic attributes of radioisotopes with specific antibodies and antibody-derived targeting agents to eliminate tumors irrespective of their location. The proof of concept for RIT was established in preclinical models during the 1970s through the development of hybridomas by Kohler and Milstein [2]. These investigations gradually transitioned into clinical applications, taking nearly 25 years. The initial focus was to demonstrate the selective targeting of cancer by antibodies, and their therapeutic potential was eventually validated. Initially, radioiodinated monoclonal antibodies (mAbs) dominated clinical trials [3].

Beta (β^−^) particles are characterized by a particle path length of up to 12 mm and a low linear energy transfer (LET, approximately 0.2 keV/μm). The Food and Drug Administration has only approved two RITs using β^−^ emitters (β-RITs) for the treatment of relapsed, refractory non-Hodgkin lymphoma, namely [^90^Y]Y-ibritumomab tiuxetan (Zevalin^®^) and [^131^I]I-tositumomab (Bexxar^®^). These RIT agents target the CD20 antigen, which is found in B-cells and B-cell malignancies. Zevalin^®^ demonstrated an 80% overall response rate (ORR) and a 30% complete response rate (CRR) compared with 56% and 16%, respectively, for the conventional treatment with rituximab, a non-radiolabeled anti-CD20 mAb therapeutic [4,5]. Bexxar^®^ also demonstrated good therapeutic efficacy, with a 95% ORR and 75% CRR [6]. However, the sale of Bexxar was discontinued, and only Zevalin is now available.

Conversely, β-RIT for solid tumors has not yet been successful in clinical practice. One of the main reasons is the lower sensitivity of solid tumors to ionizing radiation. The low ionization capacity of electrons (i.e., low LET) indicates that β^−^ radiation cannot deliver a lethal dose to a targeted cancer cell. In addition, hypoxic regions are widely observed in solid tumors, which is a major factor causing radioresistance via the “oxygen effect.” Oxygen enhances low LET radiation to indirectly induce DNA damage (indirect effect) by the generation of free radicals [7,8,9]. Moreover, the indirect effect on DNA has been shown to account for 70% of DNA damage, with 30% due to direct effects [10].

Alpha (α) particles exhibit a moderate path length (50–100 μm) and a high LET of 80 keV/μm, making them ideal for treating smaller tumor burdens, micrometastatic disease, and disseminated disease with minimal exposure to healthy tissue [11,12]. The main target of radiation is the cell nucleus; when comparing α particles to β^−^ particles, a 2–10-fold higher relative biological effectiveness can be observed [13,14]. In addition, high LET mostly induced DNA damage by interacting directly with DNA molecules, causing ionization and the formation of more complex damaged sites [10,15,16]. That has a small oxygen effect which is independent of the oxygen concentration [17]. Therefore, it is advantageous in the treatment of hypoxic tumors, such as solid tumors. The first α-emitter to obtain FDA approval was radium-223 dichloride. It showed very promising results for prostate cancer with metastatic bone lesions. Thus, targeted alpha therapy (TAT), employing α-emitters, has gained significant popularity in recent decades as a new approach [18].

Over roughly the past decade, many studies have evaluated RIT labeled with actinium-225 for cancer therapy [19,20,21]. For instance, Minnix et al. compared the effectiveness of ^177^Lu (β^−^emitter)- and ^225^Ac (α-emitter)-labeled antibodies in a murine animal model [20]. They showed that ^225^Ac outperformed ^177^Lu in delaying tumor growth and reducing overall body toxicity. These findings highlight the superior efficacy of RIT using α-emitters (α-RIT) over β-RIT while staying within the tolerance dose. The result highlights the superior efficacy of α-RIT to β-RIT within the tolerance dose [19].

Although α-RIT seems to be a promising option for cancer therapy, recent publications on α-RIT remain limited. This review provides an overview of the current state of actinium-225-, bismuth-213-, thorium-227-, and lead-212-labeled antibodies and their derivatives in cancer therapy. It also outlines the challenges with this approach and potential strategies which have high therapeutic effects.

## 2. α-Emitters for RIT

### 2.1. Actinium-225 (^225^Ac)

^225^Ac is an α-emitter with a long half-life of 9.92 days and a decay chain delivering four α and two β^−^particles. ^225^Ac is not naturally abundant and must be artificially produced through either generator-based or accelerator-based methods. The most established approach involves harvesting ^225^Ac from the decay of Thorium-229 (^229^Th), which is hampered by the low global availability of ^229^Th [22]. An alternative under development utilizes proton irradiation of Radium-226 (^226^Ra) targets in cyclotrons [23,24], offering the potential for scalable production. Following production, ^225^Ac requires radiochemical purification under high-radiation shielding conditions to ensure clinical-grade quality.

^225^Ac-labeled mAbs have gained considerable interest for α-RIT. ^225^Ac-RIT targeting cell membrane antigens such as prostate-specific membrane antigen (PSMA), insulin-like growth factor-1 receptor, carcinoembryonic antigen, CD33, CD45, and CD38 have been reported.

The application of single or multiple doses of [^225^Ac]Ac-labeled mAbs have been successful in treating xenograft models, exhibiting efficacy without inducing acute systemic toxicity [25,26]. [^225^Ac]Ac-DOTA-HuM195/lintuzumab targeting CD33 in combating blood cancer has also been reported [27]. The findings demonstrated that a single dose of [^225^Ac]Ac-DOTA-HuM195 was sufficient to induce a durable response, contrasting with a 21-day regimen of venetoclax in an acute myeloid leukemia model [27]. McDevitt et al. examined [^225^Ac]Ac-DOTA-hu11B6 for prostate cancer. Studies have revealed that ^225^Ac-labeled mAbs effectively eradicate androgen receptor-addicted prostate cancer cells [18,19].

The initial first-in-human trial demonstrated that single doses of [^225^Ac]Ac-J591 in 32 patients with pretreated, progressive, metastatic, castration-resistant prostate cancer (mCRPC) were generally well-tolerated, and the primary dose-limiting toxicity was hematologic (blood-related), such as thrombocytopenia and neutropenia. Accordingly, the maximum tolerated dose was determined to be 93.3 kBq/kg. Moreover, early signs of antitumor activity were observed, with some patients showing a reduction in prostate-specific antigen levels, suggesting potential efficacy in targeting PSMA-positive cancers. These toxicities were dependent on the dose and were manageable with dose adjustments [28].

Overall, the utilization of ^225^Ac-labeled mAb therapy in treating cancer has yielded promising outcomes across diverse studies, showcasing its efficacy in addressing various cancer types. The current quantity of available ^233^U/^229^Th is only enough to produce approximately 67 GBq (1.2 Ci) of ^225^Ac per year, yet the estimated demand exceeds 1850 GBq (50 Ci) per year [29]. This limitation leads to uncertain availability of ^225^Ac and increased costs and restricted research opportunities. Consequently, addressing this shortage through alternative production routes has become a priority.

### 2.2. Bismuth-213 (^213^Bi)

**Figure 1:**
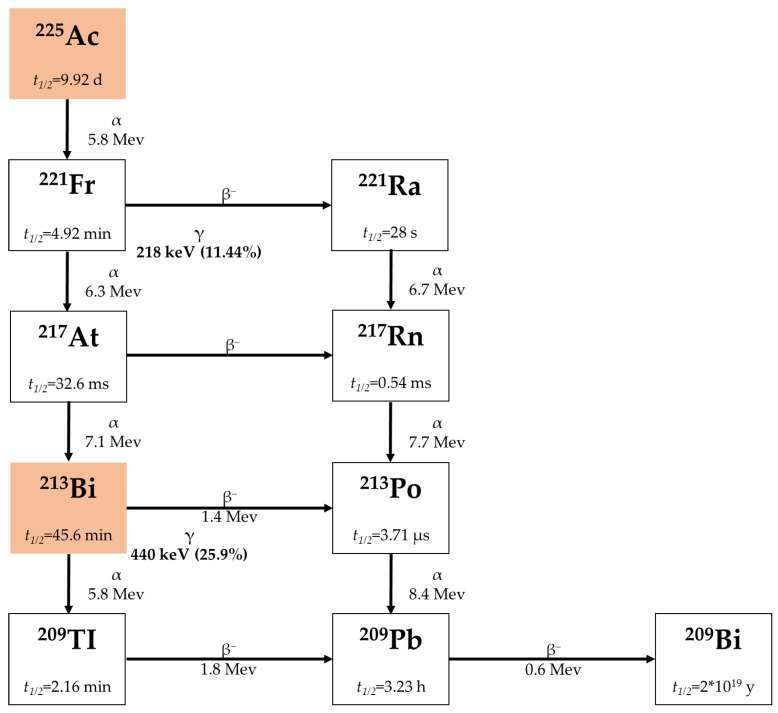
Decay chain of ^225^Ac and ^213^Bi [36].

^213^Bi is one of the commonly used α-emitters for TAT. Figure 1 shows a half-life for ^213^Bi of around 46 min and it decays to short-lived α-emitter polonium-213 (t_1/2_ = 4.2 μs, Eα = 8.37 MeV) via β^−^emission (E_β_*^−^*, ave = 435 keV, 98%). ^213^Bi also decays through α-emission to thallium-209 (^209^Tl) [30].

^213^Bi-labeled mAbs have been employed in numerous preclinical and clinical studies across various oncologic conditions because of its short half-life, such as by intravenous (i.v.) administration for hematologic cancer or intraperitoneal (i.p.) administration for peritoneal dissemination [31,32,33]. In localized treatment, because any unbound radiolabeled mAbs gradually enter systemic circulation and cause hematotoxicity, short half-life radionuclides are suitable [34].

In a clinical investigation, nearly all [^213^Bi]Bi-CHX-A”-DTPA-HuM195 particles promptly localized to and remained in leukemia-affected areas [35]. This study underscores the safety, feasibility, and antileukemic efficacy of [^213^Bi]Bi-CHX-A”-DTPA-HuM195 [32]. However, this product was withheld due to its very short half-life (46 min), and the need for an onsite generator has limited its utility. Therefore, it has been replaced by an analogue compound labeled with ^225^Ac, [^225^Ac]Ac-DOTA-HuM195.

### 2.3. Thorium-227 (^227^Th)

**Figure 2:**
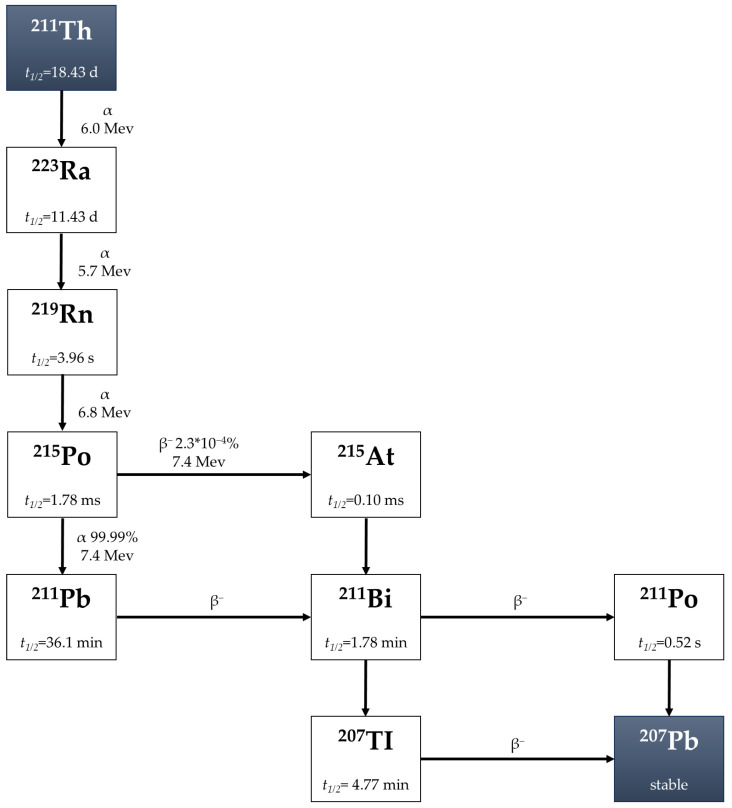
Decay chain of ^227^Th [36].

^227^Th is a promising α-emitter for employing antibodies. ^227^Th decays (E = 5.0 MeV, t_1/2_ = 18.7 days) to radium-223 (^223^Ra) through α-emission [37]. ^223^Ra (t_1/2_ 11.43 d) undergoes a decay series to stable lead-207 (^207^Pb) by four α and two β^−^ emissions, where they both also emit gamma (γ) rays, which is useful for diagnostic single-photon emission computed tomography [38]. The decay chain is shown in Figure 2. ^227^Th is primarily derived from the decay of ^231^Pa or accelerator-based production, and is commercially supplied by a limited number of manufacturers.

In a phase I clinical study, the dose-limiting toxicity of [^227^Th]^227^Th-epratuzumab was observed at 4.6 MBq. Febrile neutropenia and thrombocytopenia were observed in one patient. According to the LUGANO 2014 criteria [39], the objective response rate (ORR) was 255 (5/21 patients), including one complete and four partial responses. Thus, it was safe when it was applied in patients with relapsed or refractory B-cell non-Hodgkin lymphoma [40,41]. Another clinical development of [^227^Th]^227^Th-labeled mAbs is [^227^Th]Th-corixetan-anetumab. It has completed a phase I clinical study, yet no peer-reviewed clinical results (dose, safety, or efficacy data) have been published up to now [42].

### 2.4. Astatine-211 (^211^At)

**Figure 3:**
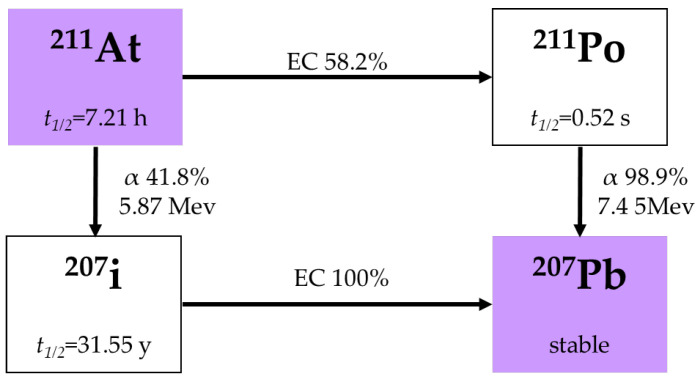
Decay chain of ^211^At [36].

^211^At is among the most widely used α-emitters in therapeutic applications. It is produced by the high-energy (25–30 MeV) cyclotron from α-particle bombardment to ^209^Bi [43] or electron capture (EC) to ^211^Po followed by α decay to stable ^207^Pb (See Figure 3) [44]. ^211^At with a half-life of 7.21 h delivers localized cytotoxicity with minimal long-lived radioactive byproducts [45]. In contrast to the radiometals listed in this chapter, ^211^At can be covalently bound to various molecules, including antibodies, peptides, and small molecules. This potential allows for greater flexibility in targeting compounds, particularly those whose binding might be affected by the chelate structure [34]. Production of ^211^At is cyclotron-based via the ^209^Bi(α,2n)^211^At reaction, requiring on-site or near-site facilities due to logistical constraints imposed by its short half-life [46].

[^211^At]At-CD123 mAbs demonstrated decreasing tumor burdens and prolonged survival doses in mice with a CD123-positive leukemia model [47]. [^211^At]At-OKT10-B10 showed significantly noticeable tumor suppression on day 21 and survived for >100 days in tumor-bearing mice [48]. Currently, clinical trials of [^211^At]At-OKT10-B10 targeting CD38 in multiple myelomas are ongoing based on this preclinical study [49].

In addition to malignant disorders, RIT using ^211^At had been developed for allogeneic hematopoietic cell transplantation in patients with aplastic anemia and hemoglobinopathies, which resulted in the rejection of the allogeneic graft. In a canine model of presensitization, the combination of [^211^At]At-anti-CD45 mAb with total body irradiation successfully abrogated graft rejection in the canine model, demonstrating a promising strategy to combat graft rejection in patients with red cell disorders [50].

Considering its not-so-long half-life, ^211^At may be suitable for application in solid tumors by using F(ab′)_2_ fragments that are specifically taken up by tumors with faster blood clearance than whole IgG. Compared with [^211^At]At-mAbs, [^211^At]At-Mel-14 F(ab′)_2_ has been shown to localize preferentially in human glioma tumor-bearing mice and remain high in tumor uptake for more two half-lives after injection [51].

From the availability aspect, ^211^At is currently produced almost exclusively through α-particle bombardment of natural Bi [36]. However, the clinical adoption of ^211^At-based radiopharmaceuticals faces a challenge in the limited availability of accelerators capable of generating the necessary 28–29 MeV α-particle beam.

## 3. An In Vivo Generator of ^212^Bi for RIT

### Lead-212 (^212^Pb)

**Figure 4:**
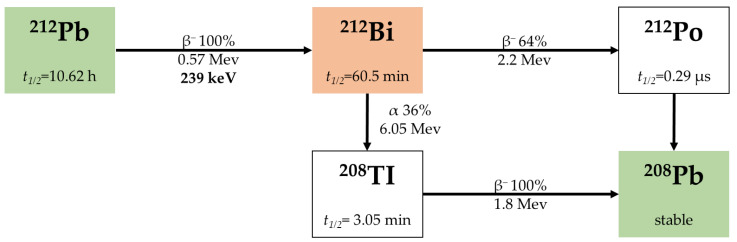
Decay chain of ^212^Pb and ^212^Bi [36].

Lead-212 (^212^Pb) is the parent of ^212^Bi and a good candidate for RIT using α-particles [40,41]. ^212^Pb is a β^−^ emitter, not an α-emitter with a half-life of 10.62 h. However, it acts as an in vivo generator decaying to α-emitters ^212^Bi (t_1/2_ = 60.5 min) and ^212^Po (t_1/2_ = 0.29 μs) [52,53,54]. In addition, ^212^Pb is well-matched in a pair of theranostics with ^203^Pb, a γ-emitter with a half-life of 51.9 h (Figure 4). Various chromatographic generator systems have been developed to isolate ^212^Pb, based on either ^228^Th or ^224^Ra as the parent nuclide [55]. Moreover, ^203^Pb can be produced through cyclotron irradiation of enriched ^203^Tl [56], which is currently available from several governmental and private sources.

[^212^Pb]Pb-TCMC-rituximab significantly decreased the tumor size and improved the survival rate in CD20-positive non-Hodgkin lymphoma mouse models. Similar results were also observed in CD38-positive multiple myeloma treated with [^212^Pb]Pb-TCMC-daratumumab [57,58]. In another study, ^212^Pb-labeled mAb YS5, targeting the CD46 epitope, successfully inhibited tumor growth and prolonged the survival of the mCRPC animal model, even with lower doses [59]. ^212^Pb-labeled trastuzumab targeting HER2-positive cancer successfully entered a phase I clinical trial and showed low agent-related toxicity [60,61].

The use of ^212^Pb is increasingly being considered in targeted α therapy due to its several advantages over ^225^Ac. One of the key benefits of ^212^Pb is its shorter half-life, which significantly reduces the clinical burden associated with prolonged patient hospital stays and complex radioactive waste management [36]. This makes ^212^Pb a more practical option for both healthcare providers and patients. Given these advantages, it is important to consider enhancing the production and availability of ^212^Pb to meet growing clinical demands.

## 4. Availability and Production Cost Analysis

Table 1 provides a comparative overview of clinically relevant α-emitting radionuclide-labeled antibodies, detailing availability, production logistics, and associated treatment costs. Particularly for ^225^Ac, the clinical scalability is constrained by high production costs and limited global supply chains. By contrast, ^212^Pb exhibits a favorable balance of radiophysical properties, logistics, and costs. Its intermediate half-life (10.6 h) enables centralized labeling and shipment, while its in vivo decay to ^212^Bi facilitates potent α-emission at the tumor site. Despite the challenge of daughter recoil and the need for optimized chelation strategies, ^212^Pb remains the most pragmatically viable isotope for broader clinical implementation, with estimated treatment costs an order of magnitude lower than those of ^225^Ac.

Meanwhile, ^213^Bi and ^211^At present distinct trade-offs: the former is limited by generator complexity and short half-life, while the latter offers favorable decay characteristics but is restricted by cyclotron availability. Taken together, these comparisons suggest that while all isotopes show therapeutic promise, ^212^Pb-labeled antibodies currently represent the most cost-effective and operationally scalable option for widespread clinical application.

## 5. Preclinical and Clinical Study

The most suitable radionuclide is chosen depending on the biological characteristics of the specific targeting vector, such as biodistribution and biological half-life [62]. In RIT, the physical half-life of the radionuclide should have an adequate half-life and must match the in vivo slow pharmacokinetics of the mAbs. If the half-life is too short, the radioactivity nearly disappears because of decay before the mAb reaches the tumor [63,64,65]. In addition, radionuclides should have an efficient production route for medical applications [36,66]. Many α-RIT studies for cancer therapy have been performed, and preclinical and clinical studies of radionuclide’s are summarized in Table 2 and Table 3, respectively. The characteristics of each α-emitter and examples of their use are also described.

## 6. Antibody Labeling

The stability of the radionuclide–antibody conjugate in vivo is important to reduce the side effects of unnecessary irradiation by the released radionuclides. This is more important for high LET α-emitters. Radiometals cannot conjugate directly to antibodies; thus, chelators are required. Although radiohalogens can conjugate directly to antibodies, labeling methods have been developed considering stable conjugates. Each labeling method is described below.

### 6.1. Radiometal Labeling

**Figure 5:**
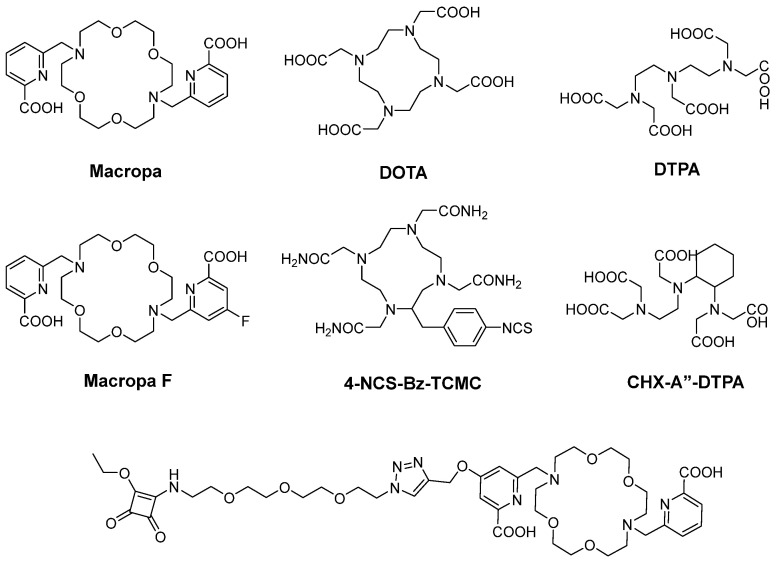
Structures of bifunctional chelating agents (BFCA) used for α-RIT and its derivatives.

The high stability of the chemical conjugation of radiometals with their targeting vector is mediated by a bifunctional chelating agent (BFCA) (Figure 5) [99,100,101]. BFCA comprises two entities: one can complex the radiometal, and the other site binds to targeting vectors including antibodies. This chelator should demonstrate high thermodynamic stability and kinetic inertness in vivo to effectively control the delivery of α-radiation to the intended target site. The BFCA type greatly influences biodistribution and in vivo stability [102].

DOTA is one of the most common chelators for radiometal labeling. High temperatures (80–95 °C) or microwave assistance are preferred for the formation of radiometal–chelate complexes [103]. However, if the reaction occurs under mild conditions, a slow kinetic labeling process ensues. Nonetheless, considering that antibodies are biomolecules, such a high reaction temperature will seriously alter the secondary and tertiary structures of the antibody and should be avoided to preserve antibody integrity [104]. Therefore, DOTA-mAbs labeled with radiometals should be reacted in <50 °C and they must be incubated for 30 min to 2 h [105,106,107]. However, DOTA is one of the most common chelators for using α-RIT, particularly ^225^Ac.

^225^Ac is predominantly chelated using the derivatives of macropa (mcp). This class of chelators, based on diaza-18-crown-6, forms exceptionally stable actinium complexes and shows higher theranostic performance than DOTA [108]. These chelators demonstrate remarkable labeling efficiency even at low concentrations, with rapid labeling at pH 7 achieved within 1 min at room temperature [74,109,110,111], making them highly suitable for sensitive biomacromolecules such as antibodies [112]. Morgan et al. reported that, in an animal experiment, MacropaSq, rather than DOTA, as a chelator for ^225^Ac led to reduced accumulation of unchelated ^213^Bi, a daughter radionuclide, in the kidneys. Thus, the body weight loss with [^225^Ac]Ac-MacropaSq-hG250 was slight compared with the loss with [^225^Ac]Ac-DOTA-hG250 at a similar dose [74,113].

Because of the high recoil energies of the α-emitting daughters, the DOTA complex of ^212^Bi, the daughter of ^212^Pb and ^225^Ac, is unstable. ^212^Bi has been reported to be lost from the ligand around 30–36% of the time [114,115]. Thus, another chelating agent has been used, i.e., 2-(4-isothiocyanotobenzyl)-1,4,7,10-tetraaza-1,4,7,10-tetra-(2-carbamoyl methyl) cyclo-dodecane (4-NCS-Bz-TCMC), which has high stability (it was reported that only 16% of ^212^Bi is released) and good efficiency and has been developed for ^212^Pb labeling with mAbs [116,117]. The optimal labeling condition of TCMC-labeled ^212^Pb is obtained at a pH of 5–6 and incubation for 15–60 min at 37 °C [59,118].

Most studies involving ^213^Bi-labeled mAbs predominantly employ CHX-A″-DTPA over DTPA or DOTA because CHX-A″-DTPA demonstrates excellent in vitro and in vivo stability and can be radiolabeled under mild conditions, preserving the immunoreactivity of the resulting conjugate. The [^213^Bi]Bi-CHX-A″-DTPA complex has exhibited notably enhanced stability compared with [^213^Bi]Bi-DTPA [119]. Another study found that achieving quantitative yields for ^213^Bi-labeling necessitates a high DOTA concentration (10 µM), whereas for CHX-A″-DTPA, 1 µM generally suffices [120]. The relatively short half-life of ^213^Bi necessitates rapid radiolabeling, leading to significant radioactivity loss due to decay, which is not the case with DOTA. The conjugation of ^213^Bi with CHX-A″-DTPA-mAbs typically occurs for 5–10 min in pH 4–5 at room temperature. To stabilize the ^213^Bi-radioimmunoconjugate, 20% ascorbic acid (pH 6.0) can be added [32,121,122]. Under these conditions, [^213^Bi]Bi-CHX-A″-DTPA-mAb has already entered clinical studies [32,121].

### 6.2. Labeling with Radiohologens

Although antibodies can be directly labeled with ^211^At, indirect labeling is used due to the instability of the direct ^211^At-protein bond [123]. Indirect labeling using prelabeled prosthetic agents, such as N-succinimidyl 3-[^211^At]At-astatobenzoate (SAB), has been extensively explored for conjugation to lysine residues [123]. However, applying this approach in clinical studies has several limitations, particularly concerning radiolabeling challenges at high activity levels, which hinder determining the maximum tolerated dose [124].

For instance, clinical trials involving [^211^At]At-labeled ch-81C6 were discontinued because of difficulties in accurately executing the labeling chemistry at dose levels of ≥370 MBq. When higher activity levels were required, both the radiochemical yield of SAB synthesis and the efficiency of SAB conjugation to mAb dropped significantly. Furthermore, after the reaction of SAB with mAb, a substantial portion (40–60%) of ^211^At activity still adhered to the walls of the reaction vessel. Lastly, the immunoreactivity of the ^211^At-labeled mAb became unacceptably low. This decline in radiochemical tractability with increasing activity levels prompted further investigations into the underlying causes [125].

**Figure 6:**
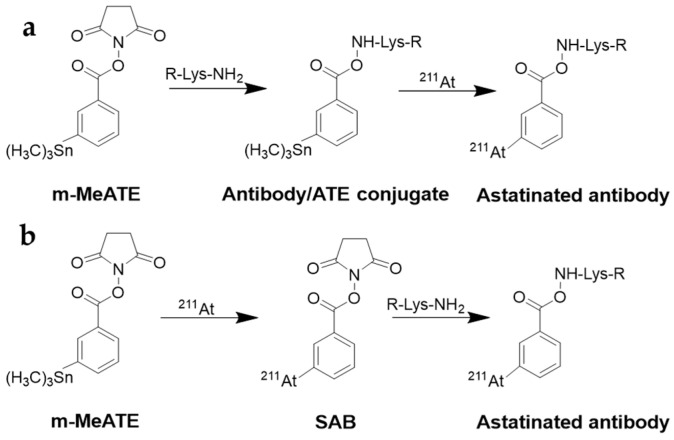
Conjugation to a lysine residue of antibody (R) and astatination. (**a**) One-step m-MeATE and (**b**) SAB conjugation method. SAB—N-succinimidyl 3-astatobenzoate.

The one-step m-MeATE method and the B10 boron cage method are currently undergoing clinical studies [49,98,123]. Compared to the conventional approach, which typically yields radiochemical yields of 30–60% within 60 min, the m-MeATE method offers significant advantages. It allows for nearly instantaneous labeling, taking only 1–20 min, with radiochemical yields ranging from 60% to 90% (Figure 6). Moreover, this method has little dependence on the concentration of the antibody conjugate, maintaining high yields even at concentrations as low as 0.125 mg/mL. In vitro stability studies in human serum demonstrated that more than 95% of the astatine was still bound to the antibody after 24 h [126].

**Figure 7:**
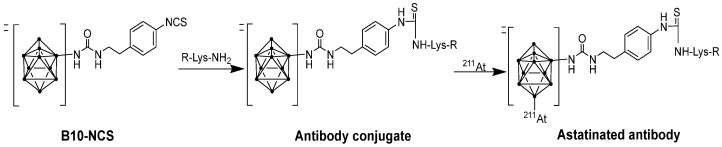
Conjugation of B10-NCS to a lysine residue of antibody (R) and astatination.

In both preclinical and clinical investigations, the boron cage compound isothiocyanatophenyl-closo-decaborate(2-) (B10) has been employed as a reagent for indirect ^211^At-labeling of mAb (Figure 7). The aromatic B10 component, with a radiochemical yield of 75–90% in just 1 min, significantly enhances ^211^At radiolabeling yield and ensures stability in vivo [85,127].

## 7. Strategies to Improve Therapeutic Effect and Reduce Toxicity

α-RIT has offered promising results in both preclinical and clinical trials, effectively eliminating or significantly reducing resistant tumors compared to conventional treatments [128]. However, IgG inherently exhibits long circulating times in the blood and low penetration into tumors [129]. The extended residence time of antibodies in the bloodstream necessitates a considerable amount of time to achieve sufficient tumor-to-background ratios following radiopharmaceutical injection. In addition to delivering the desired dose to the tumor, highly effective radiation doses are also delivered to healthy tissues, particularly the bone marrow. In addition, slow blood clearance complicates the use of α-emitters with short half-lives for the targeting of solid tumors. The large size of whole antibodies contributes to insufficient penetration in uneven drug distributions; thus, a short-range α-emitter may not irradiate the entire tumor [130,131].

In addition to enhancing the therapeutic effect, the radiation exposure of normal tissues to α-particles is still a concern in their utilization. This might be overcome by implementing a fragmented antibody, a pretargeted strategy, a local injection, or a combination strategy [61,98,132,133]. Resolving these challenges will help establish α-RIT as a general treatment approach for patients.

### 7.1. Antibody Fragments

Smaller fragment antibodies, such as minibodies, diabodies, or Fab fragments, can achieve better distribution and improve tumor penetration profiles given their reduced size and sustained specificity for the antigen [129]. For instance, [^211^At]At-A11 minibody exhibited a more uniform intertumoral activity distribution than MX35-F(ab)_2_ as shown by alpha camera imaging [87]. As mentioned previously, [^211^At]At-mAbs did not demonstrate selective uptake in the tumor compared with its fragment ([^211^At]At-Mel-14 F(ab′)_2_). [^211^At]At-Mel-14 F(ab′)_2_ can penetrate deeper into the tumor than [^211^At]At-mAbs [51].

Another study reported that [^213^Bi]Bi-CHX-A″-DTPA-C6.5K-A scFv and diabody molecules did not exert therapeutic effects in the xenograft solid tumor model. The relatively short half-life of ^213^Bi may be too brief to be effectively paired with systemically administered diabody or scFv molecules [84].

In a separate investigation, a C6.5 diabody was utilized; however, it was labeled with ^211^At, aligning the biological half-life of the delivery agent with the physical half-life of the radioisotope. The results demonstrated the effectiveness of this conjugated radionuclide as a potent agent for targeting solid tumors, indicating significant delays in tumor growth in mice. This highlights the potential usefulness of smaller antibody fragments, suitable for the half-life of each α-emitter [134].

### 7.2. Pretargeting Strategy

**Figure 8:**
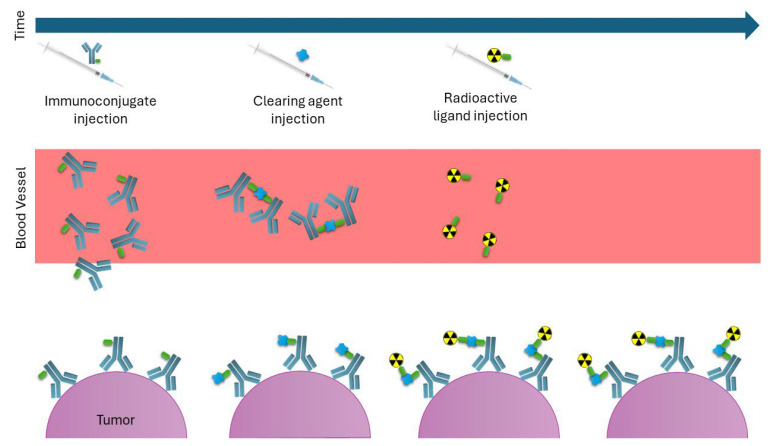
Diagram of the pretargeting strategy. First, the unlabeled antibody conjugate is injected, allowing it to accumulate slowly at the tumor. Next, a clearing agent is administered to remove any circulating antibodies from the bloodstream. Finally, the radioligand is injected. In this step, the radioligand interacts in vivo with the antibody conjugate, resulting in the formation of a radioimmunoconjugate, or it is quickly cleared from the system.

An alternative approach involves separating the antibody from the radionuclide and allowing them to combine in vivo, which is known as a pretargeting strategy. First, the antibody designed to bind both the target antigen and a radiolabeled small molecule is injected. After accumulating at the target site and clearing from the bloodstream, a complementarily radiolabeled small molecule is injected [132]. Another method involves the injection of a clearing agent to remove antibodies from the bloodstream before injecting the radiopharmaceutical (Figure 8) [135].

This pretargeting strategy offers the advantages of faster clearance of the radiopharmaceutical, thus reducing the background radiation absorbed by nontarget organs and enabling the use of short-lived radioisotopes [85,86]. Four major approaches are known as pretargeting methods: interaction between streptavidin and biotin, bispecific antibodies to bind both an antigen and a hapten, hybridization of complementary oligonucleotides, and the Diels–Alder click reaction. The details of each approach are omitted here.

Poty et al. directly compared pretargeted RIT (PRIT) and conventional RIT using ^225^Ac by assessing therapeutic efficacy and toxicity in murine models of pancreatic ductal adenocarcinoma (PDAC). The findings demonstrate the effective delivery of radioactive payloads to tumor sites with a minimized average absorbed dose in healthy tissues. This approach leads to prolonged survival and reduced hematotoxicity in subcutaneous and orthotopic PDAC models when compared with conventional RIT [132]. These results also indicate that antibody molecules gradually bind until they reach the highest concentration in tumor cells, while a clearance process occurs in normal tissue. As a result, radiolabeled small molecules injected afterwards can bind and maximally penetrate into the tumor, where they specifically bind to prelocalized antibodies that have already bound [132].

### 7.3. Local Injection/Infusion

Further strategies to enhance the targeted delivery of radiopharmaceuticals and minimize systemic toxicity involve local infusion. Consequently, blood radiation-absorbed doses do not hinder achieving the therapeutic dose when radiopharmaceuticals are administered intracompartmentally [136]. For example, patients in clinical remission following salvage chemotherapy for peritoneal recurrence of ovarian cancer underwent i.p. infusion of [^211^At]At-MX35 F(ab′)_2_ [98]. Before that, patients were given potassium perchlorate to block astatine uptake by the thyroid. The results showed that the antibodies most likely bind to the tumor cells in micrometastases through the peritoneal fluid rather than through vascular flow. Therefore, the activity concentration of [^211^At]At-MX35 F(ab′)_2_ in the peritoneal fluid determines the irradiation of the microscopic peritoneal tumors. This treatment showed potential as a well-tolerated therapy for locally confined microscopic ovarian cancer [137].

Another study showed that less than 4 h after administration of a normal tissue blocking i.v. dose of trastuzumab (4 mg/kg i.v.), patients with peritoneal carcinomatosis who had failed standard therapies underwent i.p. infusion of [^212^Pb]Pb-TCMC-trastuzumab. It showed limited redistribution of radioactivity from the peritoneal cavity to the circulating blood, and no specific uptake in major organs was observed within 24 h. In addition, it showed a lack of substantial toxicity [61,133].

### 7.4. Combination of Pretargeted Therapy with Local Injection

A pretargeting strategy with direct administration to the tumor site showed promising results in terms of efficacy and safety. A study of a pretargeting strategy through i.p. injection was implemented in PRIT with ^225^Ac-labeled anti-HER2 antibody for peritoneal carcinomatosis of epithelial ovarian cancer (EOC PC). This study also used a clearing agent. The result exhibited an effective radioactive dose delivered to the tumor with reduced harm to nontargeted tissues and prolonged survival with minimal toxicity. Although this study was simulated in preclinical studies, it might be significant for patients with EOC PC, as future studies focusing on refining targeting methods and enhancing therapeutic effects pave the way for clinical applications [71].

### 7.5. Theranostics

Numerous theranostic approaches for cancer imaging and therapy are being advanced by modifying radiolabeled antibodies through the replacement of imaging radionuclides with therapeutic radionuclides. These theranostics using radionuclides are sometimes referred to as radiotheranostics [138,139].

Since α-particles do not penetrate deeply into tissues, direct imaging is challenging. Therefore, it is desirable to use γ-emitting or positron-emitting radionuclides as imaging surrogates for α-RIT in theranostic pairs to determine radioactivity levels within tissues and organs [140]. The benefits of theranostics include the ability to perform SPECT or PET imaging prior to treatment, enabling evaluation of target antigen expression levels and facilitating screening of suitable patients. Furthermore, by using imaging radionuclides with properties similar to therapeutic α-emitters, it is possible to predict the biodistribution of therapeutic α-particles during treatment and contribute to dosimetry [138]. Specifically, theranostic approaches are being developed using identical radionuclide pairs such as ^203^Pb/^212^Pb for melanoma [141], as well as theranostics using radionuclide pairs with similar properties, including ^225^Ac/^134^Ce for CD46 [108], ^227^Th/^89^Zr for CD20 [142], ^211^At/^89^Zr for GPC1 [143], and ^225^Ac/^111^In for HER2 and MUC1 [144,145]. The theranostics concept can also be effective when combined with the previously mentioned antibody fragment and pretargeting methods, and a pretargeted α-RIT with ^203^Pb/^212^Pb theranostic pair has been investigated [146].

Additionally, radiopharmaceuticals with identical molecular structures can be utilized for both imaging and therapy through the macropa-F ligand. For instance, the [^18^]macropa-F ligand can be used for PET imaging, while ^225^Ac, ^212^Pb, or ^213^Bi labeled with macropa-F can be used for therapy [147]. Although macropa-F ligands have not yet been studied in preclinical or clinical trials, they show promise for ensuring matched pharmacokinetic profiles. We expect that the matching may facilitate accurate treatment planning and enhance therapeutic precision.

## 8. Future Prospects

**Figure 9:**
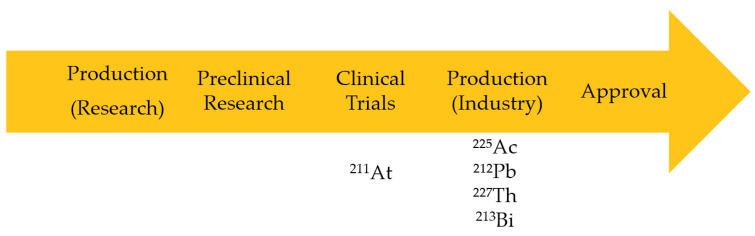
Schematic representation of the α-RIT bench-to-bedside progression of each α-emitter, from production (research) to radiopharmaceutical approval [148].

α-RIT has shown superior results to β-RIT, potentially altering the treatment paradigm for several cancer indications; however, publications on this topic remain scarce. Further research and development on α-RIT is expected, and several issues remain to be addressed. Firstly, the global availability of α-emitters remains limited. Notably, radioimmunoconjugates utilizing α-emitters such as ^225^Ac, ^212^Pb, ^213^Bi, and ^227^Th have progressed to production stages, supported by commercial sponsors including pharmaceutical companies (Figure 9). α-RITs such as [^225^Ac]Ac-DOTA-HuM195/lintuzumab, [^225^Ac]Ac-DOTA-hu11B6, [^225^Ac]Ac-FPI-1434, [^213^Bi]Bi-CHX-A”-DTPA-HuM195/lintuzumab, [^225^Ac]Ac-macropa-pelgifatamab, [^227^Th]Th-epratuzumab, [^227^Th]Th-corixetan-anetumab, and [^212^Pb]Pb-TCMC-trastuzumab are supported by commercial companies.

As a result, the demand for α-emitting radionuclides is expected to increase significantly in the near future. To enable widespread clinical implementation, continued industrial investment and infrastructure development for α-emitter production will be essential to meet the growing demand for these promising α-RITs. Moreover, the availability of suitable α-particle emitters at reasonable costs must be addressed concurrently and the production should be scaled up effectively and efficiently.

Second, the clinical development and optimization of α-RITs face challenges regarding pharmacokinetics. A key difficulty lies in identifying the optimal half-life of therapeutic radionuclides. This optimal window must balance biological targeting time and radionuclide stability: a radionuclide with a half-life too short may decay before accumulating in the target tissue, whereas one with a half-life too long risks detaching from the pharmaceutical complex and causing off-target radiation exposure [63]. Therefore, more effort must be expended to match the physical half-lives of therapeutic isotopes with the biological properties of antibodies for α-RITs in cancer therapy [84].

To improve the therapeutic efficacy and reduce the toxicity of α-RIT, five approaches have been previously outlined, with pretargeting strategies still in relatively early investigational stages. One promising strategy involves the use of antibody fragments (e.g., nanobodies, minibodies, scFv, and diabodies) labeled with α-emitters. Compared to whole antibodies, these fragments are often more efficiently internalized into target cells [149], enabling a greater fraction of the recoiling daughter radionuclides to remain intracellular. Thus, the radiation exposure of surrounding healthy tissues is reduced. Although this strategy demonstrates promising results in preclinical and clinical studies, as we mentioned before, this approach requires careful consideration in selecting an appropriate α-emitter. So far, among the α-emitters discussed in this paper, ^211^At is the most suitable match for the biological properties of antibody fragments. This is because ^211^At has a physical half-life of 7.2 h, which aligns well with the rapid tumor targeting and clearance kinetics of these smaller antibodies [149]. Thus, we suggest that ^212^Pb may also be suitable for antibody fragments with a physical half-life of 10.62 h. Furthermore, other major challenges of this strategy are the rapid clearance and short plasma half-life of antibody fragments, which can compromise tumor uptake and lead to undesirable renal accumulation of radioactivity. To mitigate this, co-infusion with agents such as Gelofusine and/or lysine is commonly employed to competitively inhibit the reabsorption of radiolabeled fragments in the proximal tubules, thereby reducing renal uptake [68]. Moreover, the specificity and potency of treatment can be further refined by optimizing the structural design of antibody fragments. In particular, the development and implementation of site-specific conjugation strategies are critical for maximizing the safety, efficacy, and reproducibility of antibody fragment-labeled α-emitters. Such approaches ensure homogeneous conjugate populations, preserve antigen-binding affinity, and minimize off-target effects, thereby enhancing the therapeutic windows and clinical translational potential of these agents [150].

Another promising approach involves the direct administration of α-RIT into or near the tumor site, including intratumoral injection or delivery into the peritoneal cavity following tumor resection. In a phase I clinical study in ovarian cancer patients, a single i.p. infusion of [^211^At]At-MX35 F(ab′)_2_ in 1–2 L of Extraneal^®^ solution was administered. The findings demonstrated the potential to achieve therapeutic absorbed doses without inducing significant systemic toxicity. Most adverse events were low-grade and likely associated with procedural aspects rather than the radiopharmaceutical itself. Notably, one of twelve patients experienced a grade 4 intestinal perforation, which were likely related to complications from catheter placement. Due to the procedural challenges associated with i.p. catheter placement, it is essential to establish a well-defined standard operating procedure (SOP) to enhance the safety and reliability of localized radiopharmaceutical administration. Proper protocol development and strict adherence can significantly minimize the risk of complications and improve overall treatment outcomes.

In addition, dosimetry data should be systematically collected, as treatment planning and verification may help assist the deployment of radiopharmaceuticals and could guide the optimal prescribed activity in clinical studies. The doses absorbed by each organ can be assessed through pharmacokinetic modeling from quantitative imaging or radioactivity measurement in blood samples based on the International Commission on Radiological Protection (ICRP) model [151]. For instance, administration of ^223^Ra-Chloride to the human body with an equivalent of 21 MBq for 70 kg resulted in an absorbed α dose to the bone endosteal cells and the red bone marrow of about 16 Gy and 1.5 Gy, respectively [152]. The use of imaging surrogates with chemical properties more closely resembling those of therapeutic α-emitters has been proposed. As previously discussed, theranostic approaches utilizing radionuclide pairs with similar physical and chemical characteristics or radiopharmaceuticals with identical molecular structures for imaging and therapy are being explored.

Another challenge when dealing with α-emitting radionuclides that have complex decay chains—such as ^225^Ac, ^212^Pb, or ^227^Th—is that it is essential to account for the behavior of all daughter radionuclides. These decay products may contribute to the therapeutic effect, but they can also lead to off-target toxicity if not properly controlled. Particular attention must be paid to the retention of daughter nuclides within chelate complexes [33,36,74]. To address this, chelators suitable for α-RIT are being developed with improved stability to minimize the release of radioactive daughters post-decay, while also enabling efficient radiolabeling under mild conditions. Furthermore, due to the presence of radioactive decay products, rigorous and rapid quality control (QC) procedures are crucial to ensure labeling efficiency and radiochemical purity prior to clinical application.

Another major challenge in the clinical translation of α-RIT is the significant investment required in infrastructure. In particular, the high-energy γ-rays emitted by the ^208^Tl daughter of ^212^Pb necessitate enhanced shielding measures, which substantially increase the overall costs of radiopharmacy installations and patient administration facilities. Furthermore, the short half-lives of α-emitters, such as ^211^At and ^213^Bi, present logistical limitations for centralized production. To mitigate this, the strategic distribution of particle accelerators for ^211^At or an onsite generator for ^213^Bi closer to clinical sites has been proposed, minimizing the need for long-distance transport and enabling more timely and efficient delivery of short-lived radioisotopes.

Therefore, interdisciplinary collaboration is essential for conducting research in this field. Consequently, numerous preclinical studies are expected to translate into clinical research with favorable outcomes for patients in the future.

## 9. Conclusions

α-RIT presents a unique opportunity for tumor treatment by employing α-emitters guided to tumor sites using targeting agents with specific targets and low toxicity to surrounding normal tissues. The advantageous characteristics of α-emissions, including higher linear energy transfers and shorter path ranges, contribute to increased therapeutic effectiveness with a reduced risk of side effects compared with other radiation types. Despite promising clinical outcomes, widespread clinical adoption is currently limited by several key challenges: the restricted global supply and high production cost of clinically relevant α-emitters, the need for optimized radionuclide half-life matching with the pharmacokinetics of targeting vectors, and the requirement for improved chelators to retain daughter nuclides in complex decay chains. Ongoing strategies such as antibody fragmentation, pretargeting strategies, local injection, or combination approaches are in development and show promise for improving therapeutic outcomes and minimizing toxicity. Several promising α-RIT agents are currently being evaluated which will offer potential alternative therapies for treating malignancies, particularly in cases where other therapeutic options are limited or not feasible.

Maximizing the therapeutic benefits of alpha-radiation immunotherapy (α-RIT) may require collaborative efforts to improve the infrastructure for isotope production, develop advanced chelation techniques, and refine delivery methods tailored to specific types of cancer.

## Figures and Tables

**Table 1:** Highlights of the production cost and availability of α-emitting radionuclides [36].

Isotope	Production Cost	Availability
^225^Ac	High	Limited (1:42; production: demand) but expanding
^213^Bi	High	Limited
^227^Th	Low	Available
^211^At	Low	Limited to a few centers
^212^Pb	Low	Available

**Table 2:** Overview of some preclinical trials on alpha radioimmunotherapy (α-RIT).

TAT Agent	Target; Mouse Model	Findings	Route and Activity	Ref.
[^225^Ac]Ac-DOTA-BC8	CD45; multiple myeloma	Selective killing of CD45^+^ and safe, targeted conditioning for bone marrow transplants.	N/A; a single dose of 300 nCi	[67]
[^225^Ac]Ac-DOTA-2Rs15d sdAb	HER2; HER2-expressing cancer	Significantly extending survival compared to control and to trastuzumab alone. Renal toxicity is found.	i.v.; 3 × 85 kBq	[68]
[^225^Ac]Ac-DOTA-HuM195/lintuzumab + venetoclax	CD33; acute myeloid leukemia	Combination showed superior tumor control and significantly prolonged survival in venetoclax-resistant.	7.4 kBq (i.p.) + 200 mg/kg venetoclax (orally)	[27]
[^225^Ac]Ac-DOTA-hu11B6	hK2; prostate cancer	Single high dose is better. No treatment-related toxicity observed.	i.v.; single high dose 22 kBq or 2 × ~11.1 kBq (300 nCi) spaced 4.5 months	[25,69]
[^225^Ac]Ac-DOTA-PKU525	FAP; cancer-associated fibroblast	Significant tumor inhibition. No weight loss or organ damage.	i.v.; single dose ~11.1 kBq (300 nCi)	[26]
[^225^Ac]Ac-DOTAylated-huCC49	TAG-72; ovarian cancer	Single high dose extended survival more than 3-fold over control. It is similar to multidose. No weight loss and no organ toxicity.	i.v.; single high dose 7.4 kBq or multi-dose 1.85 kBq followed by 5 × 0.7 kBq 5 weekly dose	[70]
[^225^Ac]Ac-anti-HER2/anti-DOTA IgG-scFv BsAb (pretargeted radioimmunotherapy)	HER2; ovarian cancer	Both single and double cycles prolonged survival. RBE-weighted dose per cycle: tumor is 56.9 Gy and kidneys are 16.1 Gy.	i.p; 37 kBq or 2 × 37 kBq (spaced 1 week).	[71]
[^225^Ac]Ac-DOTA-OTSA101	FZD10; synovial sarcoma	Reduced tumor volume and prolonged survival. 60% of mice receiving a single 7.4 kBq dose achieved a complete response with no tumor recurrence.	i.v.; 7.4 kBq	[72]
[^225^Ac]Ac-DOTA-hu5A10	FcRn; prostate cancer	Sustained tumor control. 7/18 complete remissions.	i.v.; ~11.1 kBq (300 nCi)	[73]
[^225^Ac]Ac(MacropaSq-hG250)	Carbonic anhydrase IX; renal cell carcinoma	Specific tumor targeting, significant tumor inhibition and DNA damage. Reduced kidney toxicity compared to DOTA-based variants. Fast, stable room-temperature labeling of antibodies with ^225^Ac.	i.v.; 14.8 kBq	[74]
[225Ac]Ac-E4G10	Cadherin; glioblastoma	Increase overall survival.	i.v.; 11.1 kBq (300 nCi)	[75]
[^213^Bi]Bi-CHX-A″-DTPA-anti-EGFR-mAb	EGFR; bladder carcinoma	Both dosing regimens yielded potent antitumor effects with durable responses in over 30% of subjects. No normal tissue toxicity.	i.p; 2 × 0.93 MBq or 3 × 0.46 MBq	[76]
[^213^Bi]Bi-CHX-A″-69-11 antibodies	CETN1; pancreatic ductal adenocarcinoma	Significant tumor suppression. No weight loss or organ damage observed.	i.p.; no specified	[77]
[^213^Bi]Bi-CHX-A″-h8C3 antibody + anti-PD-1	Melanin; melanoma	The combination showed significant tumor control and prolonged survival. No weight loss or over toxicity.	i.p.; ~0.46–0.93 MBq (1–2 doses)	[78]
[^213^Bi]Bi-CHX-A″-DTPA-MX35-mAb	NaPi2b; ovarian cancer	The tumor-free rates for low and high doses are 55% and 78%, respectively. No weight loss, stable WBC/platelet.	i.p.; 3 MBq/mL (~10 µg) or 9 MBq/mL (~30 µg)	[31]
[^213^Bi]Bi-DTPA-PAN-622-mAb	HAAH; breast cancer	Significant inhibition of primary tumor growth.	i.p.; 150 μCi	[79]
[^213^Bi]Bi-CHX-A″-DTPA-Anti-hCD138 antibody	CD138; ovarian cancer	Single i.p. injections of both 7.4 and 11.1 MBq doses significantly prolong survival.	i.p.; 7.4 MBq or 11.1 MBq	[80]
[^213^Bi]Bi-DOTA-9E7.4	CD138; multiple myeloma	Median survival increased to 80 days vs. 37 days in control group and 54 days in 18.5 MBq [^177^Lu] Lu-DOTA-9E7.4. ~45% of mice were cured, exhibiting long-term complete remission.	i.v.; 3.7 MBq	[81]
[^213^Bi]Bi-CHX-A″-DTPA-anti-CD138-mAb + adoptive T cell therapy	CD138; multiple myeloma	The combination achieved a significant tumor growth delay compared to either treatment alone.	i.v.; 3.7 MBq followed by 5 × 10^6^ T cells injection after 24 h.	[82]
[^213^Bi]Bi-DTPA-anti-CD38-mAb	CD38; multiple myeloma	Dramatic tumor suppression and significantly extended survival.	i.v.; 6 × 1.85 MBq	[83]
[^213^Bi]Bi-CHX-A″-DTPA-C6.5K-A scFv and diabody	HER2; ovarian cancer	The 0.3 µCi dose of scFv resulted in a significant reduction in tumor growth rate compared to controls. Acceptable toxicity levels. However, it was not antigen specific. Diabody conjugates did not significantly inhibit tumor growth compared to controls.	i.v.; diabody: 0.64, 0.35, and 0.15 µCi scFv: 1.1, 0.6, and 0.3 µCi	[84]
[^211^At]At-anti-CD123-mAb	CD123; leukemia	Decreased tumor burdens and substantially prolonged survival.	i.v.; 40 µCi	[47]
[^211^At]At-OKT10	CD38; multiple myeloma	Sustained remission and long-term survival (>150 days) for 50% to 80% of treated mice.	i.v.; 24 to 45 µCi	[48]
[^211^At]At-CA12.10C12 + total body irradiation (TBI)	CD45; aplastic anemia and hemoglobinopathy	The combination was successful in abrogating graft rejection in 86% of dogs in this presensitization model.	i.v.; 0.188 mCi/kg (7 MBq) on day-3, and TBI followed by marrow grafts on day 0.	[50]
[^211^At]At-1F5-B10	CD20; minimal residual disease lymphoma	Complete eradication of disseminated lymphoma in treated mice, with no detectable disease at 90 days post-treatment. [^211^At]At-1F5-B10 demonstrated superior therapeutic efficacy compared to its ^131^I-labeled counterpart.	i.v.; up to 0.5 mCi/kg	[85]
[^211^At]At-9E7.4	CD 138; multiple myeloma minimal residual	The 740 kBq significantly prolonged survival, with about 65% of mice surviving at 150 days post-treatment.	i.v.; 370, 555, 740, and 1100 kBq	[86]
[^211^At]At-Mel-14 F(ab′)_2_	Chondroitin sulfate proteoglycan; gliomas	Specifically localized to human glioma xenografts in mice. Good tumor uptake and retention.	i.v.; N/A	[51]
[^211^At]At-A11	PSCA; prostate cancer or bone microtumors	Lower doses showed efficacy with minimal toxicity.	i.v.; 0.3 to 1.0 MBq	[87]
[^212^Pb]Pb-TCMC-rituximab	CD20; non-Hodgkin lymphoma	Significantly prolonged median survival compared to controls. Toxicity was dose-dependent; lethal effects occurred at doses exceeding 740 kBq. At 277.5 kBq, the treatment was well tolerated with minimal hematological toxicity.	i.v.; 277.5 kBq	[57]
[^212^Pb]Pb-TCMC-daratumumab	CD38; multiple myeloma	Efficacy at 277.5 kBq without toxic effects.	i.v.; 185kBq or 277.5 kBq	[58]
[^212^Pb]Pb-TCMC-YS5	CD46; prostate cancer	0.74 MBq effectively and safely inhibited tumor growth and enhanced survival.	i.v.; 0.74 MBq	[59]
[^212^Pb]Pb-TCMC-NNV003	CD37; chronic lymphocytic leukemia and non-Hodgkin lymphoma	Daudi model (CB17 SCID): 67–91% survival at 28 weeks post-cell injection. MEC-2 model (R2G2): 30–90% survival at study endpoint (~21 weeks). Mild/transient hematology effects; no major organ toxicity.	i.v.; 185–555 kBq	[88]

Abbreviations: N/A—not available; CD—cluster of differentiation; HER2—human epidermal growth factor receptor 2; hK2—human kallikrein-2; FAP—fibroblast activation protein; TAG-72—tumor-associated glycoprotein 72; FZD10—frizzled homolog 10; FcRn—neonatal Fc receptor; EGFR—epidermal growth factor receptor; CETN1—centrin 1; HAAH—human aspartyl (asparaginyl) β-hydroxylase; PSCA—prostate stem cell antigen.

**Table 3:** Overview of some clinical trials on alpha radioimmunotherapy (α-RIT).

TAT Agent	Target, Indication	Route and Activity	Status	Findings	Ref.
[^225^Ac]Ac-DOTA-HuM195/lintuzumab + venetoclax	CD33; acute myeloid leukemia	i.v.; 18.5 or 9.25 kBq/kg on day 5 (4 cycles) + Venetoclax on day 1–21 (12 cycles)	Phase I/II, recruiting (2020)	Recruiting, not yet reported.	[89]
[^225^Ac]Ac-FPI-1434	IGF-1R; advanced solid tumours	N/A; dose is per cohort assignment.	Phase I/II, recruiting (2019)	Recruiting, not yet reported	[90]
[^225^Ac]Ac-J591	PSMA; mCRPC	i.v.; 65 or 50 kBq/kg	Early phase I, active, not recruiting (2020)	Not yet reported	[28,91,92,93]
		i.v.; single dose every 6 weeks × 4	Phase I/II, suspended (2020)	Not yet reported	
		i.v.; 13.3–93.3 or 0.36–2.52 kBq/kg on day 1	Phase I, completed (2017)	Dose-limiting toxicity was 80 KBq/kg and the recommended phase II dose was 93.3 KBq/kg	
[^225^Ac]Ac-DOTA-M5A	CEA; positive colorectal cancer	i.v.; over 25 min on day 1, dose is per cohort assignment.	Phase I, recruiting (2022)	Not yet reported	[94]
[^225^Ac]Ac-DOTA-daratumumab + fludarabine + melphalan + total marrow and lymphoid irradiation (TMLI)	CD38; high-risk myeloid leukemia, acute lymphoblastic leukemia, and myelodysplastic syndrome	i.v.; injection on day 15. The dose is per cohort assignment. TMLI BID on days −8 to −5, fludarabine IV on days −4 to −2, and melphalan IV on day −2, followed by HCT on day 0.	Phase I, recruiting (2024)	Not yet reported	[95]
[^225^Ac]Ac-DOTA-hu11B6	hK2; advanced prostate cancer	i.v.; one or multiple doses. The dose levels will be escalated based on the dose-limiting toxicities.	Phase I, recruiting (2020)	Not yet reported	[96]
[^225^Ac]Ac-macropa-pelgifatamab	PSMA; mCRPC	i.v.	Phase I, recruiting (2023)	Not yet reported	[97]
[^213^Bi]Bi-CHX-A”-DTPA-HuM195/lintuzumab	CD33; acute myeloid leukemia	i.v; 18.5, 27.75, 37, and 46.25 kBq/kg	Phase I/II, completed (2001)	MTD = 37MBq/kg. Treatment-related deaths occurred = 10% of those who received the MTD.	[32]
[^227^Th]Th-corixetan-anetumab	Mesothelin; malignant pleural epithelioid, malignant peritoneal epithelioid, and ovarian cancer	i.v.; 1.5 MBq	Phase I, completed (2018)	Not yet reported	[42]
[^211^At]At-MX35 F(ab′)_2_	95-kDa plasma membrane sodium-dependent phosphate transporter protein 2b (NaPi2b); ovarian	i.p. infusion; dose escalation up to 215 MBq/L (5MBq/kg)	Early phase I, completed (2005)	i.p. administration is possible to achieve therapeutic absorbed doses without significant toxicity.	[98]
[^211^At]At-OKT10-B10 + fludarabine	CD38; high-risk multiple myeloma	i.v.	Phase I, not yet recruiting (2024)	Not yet reported	[49]
[^212^Pb]Pb-TCMC-trastuzumab	HER2; HER2-expressing malignancies in the peritoneal cavity	i.p.; dose escalation up to 40 MBq	Phase I, completed (2011)	MTD = 27 MBq/m^2^ = 0.9 MBq/kg	[61]
[^227^Th]^227^Th-epratuzumab	CD22; relapsed/refractory CD22-positive non-Hodgkin lymphoma	i.v.; dose up to 4.6 MBq	Phase I, completed (2015)	Tolerated dose up to 4.6 MBq (10 mg antibody) without reading MTD. Safe and tolerated in patients with R/R-NHL.	[38]
[^227^Th]Th-corixetan-anetumab	Mesothelin; solid tumors known to express mesothelin	i.v.; start at 1.5 MBq and increase in steps of 1.0 or 1.5 MBq	Phase I, Completed (2018)	Not yet reported	[42]

The year in the clinical trial row refers to the date when the clinical study was (or is expected to be) initiated. Abbreviations: N/A—not available; CD—cluster of differentiation; IGF-1R—insulin-like growth factor-1 receptor; mCRPC—metastatic castration-resistant prostate cancer; PSMA—prostate-specific membrane antigen; CEA—carcinoembryonic antigen; R/R-NHL—relapsed/refractory B cell non-Hodgkin lymphoma; hK2—human kallikrein-2; FAP—fibroblast activation protein; MTD—maximum tolerated dose.

## Data Availability

Not applicable.
